# Mechanical ventilation and death in pregnant patients admitted for COVID-19: a prognostic analysis from the Brazilian COVID-19 registry score

**DOI:** 10.1186/s12884-022-05310-w

**Published:** 2023-01-10

**Authors:** Zilma Silveira Nogueira Reis, Magda Carvalho Pires, Lucas Emanuel Ferreira Ramos, Thaís Lorenna Souza Sales, Polianna Delfino-Pereira, Karina Paula Medeiros Prado Martins, Andresa Fontoura Garbini, Angélica Gomides dos Reis Gomes, Bruno Porto Pessoa, Carolina Cunha Matos, Christiane Corrêa Rodrigues Cimini, Claudete Rempel, Daniela Ponce, Felipe Ferraz Martins Graça Aranha, Fernando Anschau, Gabriela Petry Crestani, Genna Maira Santos Grizende, Gisele Alsina Nader Bastos, Giulia Maria dos Santos Goedert, Luanna Silva Monteiro Menezes, Marcelo Carneiro, Marcia Ffner Tolfo, Maria Augusta Matos Corrêa, Mariani Maciel de Amorim, Milton Henriques Guimarães Júnior, Pamela Andrea Alves Durães, Patryk Marques da Silva Rosa, Petrônio José de Lima Martelli, Rafaela Santos Charão de Almeida, Raphael Castro Martins, Samuel Penchel Alvarenga, Eric Boersma, Regina Amélia Lopes Pessoa de Aguiar, Milena Soriano Marcolino

**Affiliations:** 1 Department of Gynecology and Obstetrics, University Hospital. Universidade Federal de Minas Gerais, Av. Professor Alfredo Balena, 190, Belo Horizonte, Brazil; 2 Department of Statistics, Universidade Federal de Minas Gerais, Av. Presidente Antônio Carlos, 6627, Belo Horizonte, Brazil; 3 Universidade Federal de São João del-Rei, R. Sebastião Gonçalves Coelho, 400, Chanadour, Divinópolis, MG 35501-296 Brazil; 4 Department of Internal Medicine, Medical School, Universidade Federal de Minas Gerais, Av. Professor Alfredo Balena, 190, Belo Horizonte, Brazil; 5 Institute for Health Technology Assessment (IATS/CNPq), R. Ramiro Barcelos, 2359, Porto Alegre, Brazil; 6 University Hospital, Universidade Federal de Minas Gerais, Av. Professor Alfredo Balena, 190, Belo Horizonte, Brazil; 7 Hospital Nossa Senhora da Conceição and Hospital Cristo Redentor, Av. Francisco Trein, 326, Porto Alegre, Brazil; 8 Hospitais da Rede Mater Dei, Av. do Contorno, Belo Horizonte, 9000 Brazil; 9 Hospital Júlia Kubitschek, R. Dr. Cristiano Rezende, Belo Horizonte, 2745 Brazil; 10 Faculdade de Ciências Médicas de Minas Gerais, Alameda Ezequiel Dias, 275, Belo Horizonte, Brazil; 11 Hospital Santa Rosália, R. Dr. Onófre, 575, Teófilo Otoni, Brazil; 12 Mucuri Medical School, Universidade Federal dos Vales do Jequitinhonha e Mucuri, R. Cruzeiro, 01, Teófilo Otoni, Brazil; 13 Hospital Bruno Born, Av. Benjamin Constant, 881, Lajeado, Brazil; 14 Botucatu Medical School, Universidade Estadual Paulista “Júlio de Mesquita Filho” and Hospital das Clínicas da Faculdade de Medicina de Botucatu, Av. Prof. Mário Rubens Guimarães Montenegro, s/n, Botucatu, Brazil; 15 Hospital SOS Cardio, Rodovia, SC-401, 121, Florianópolis, Brazil; 16 Hospital Mãe de Deus, R. José de Alencar, 286, Porto Alegre, Brazil; 17 Hospital Santa Casa de Misericórdia de Belo Horizonte, Av. Francisco Sales, 1111, Belo Horizonte, Brazil; 18 Hospital Moinhos de Vento, R. Ramiro Barcelos, 910, Porto Alegre, Brazil; 19 Medical School, Universidade Federal de Santa Maria, Av. Roraima, 1000, Santa Maria, Brazil; 20 Hospital Metropolitano Odilon Behrens, R. Formiga, 50, Belo Horizonte, Brazil; 21 Hospital Santa Cruz, R. Fernando Abott, 174, Santa Cruz do Sul, Brazil; 22 Faculdade Integrada de Santa Maria, R. José do Patrocínio, 26, Santa Maria, Brazil; 23 Universidade Luterana do Brasil, Av. Farroupilha, 8001, Canoas, Brazil; 24 Hospital Márcio Cunha, Av. Kiyoshi Tsunawaki, 41, Ipatinga, Brazil; 25 Pontifícia Universidade Católica de Minas Gerais, R. do Rosário, 1081, Betim, Brazil; 26 Centro Universitário de Belo Horizonte, Av. Professor Werneck, 1685, Belo Horizonte, Brazil; 27 Centro de Ciências Médicas, Universidade Federal de Pernambuco, Hospital das Clínicas da Universidade Federal de Pernambuco, Av. Prof. Moraes Rego, 1235, Recife, Brazil; 28 Hospital Tacchini, R. Dr. José Mário Mônaco, 358, Bento Gonçalves, Brazil; 29 Department of Cardiology, University Medical Center Rotterdam, Doctor Molewaterplein 40, 3015 GD Rotterdam, The Netherlands; 30 Telehealth Center, University Hospital, Universidade Federal de Minas Gerais, Av. Professor Alfredo Balena 190, Belo Horizonte, Brazil

**Keywords:** COVID-19, Pregnant women, Clinical decision rules, Mortality, Artificial respiration, Prognosis

## Abstract

**Background:**

The assessment of clinical prognosis of pregnant COVID-19 patients at hospital presentation is challenging, due to physiological adaptations during pregnancy. Our aim was to assess the performance of the ABC_2_-SPH score to predict in-hospital mortality and mechanical ventilation support in pregnant patients with COVID-19, to assess the frequency of adverse pregnancy outcomes, and characteristics of pregnant women who died.

**Methods:**

This multicenter cohort included consecutive pregnant patients with COVID-19 admitted to the participating hospitals, from April/2020 to March/2022. Primary outcomes were in-hospital mortality and the composite outcome of mechanical ventilation support and in-hospital mortality. Secondary endpoints were pregnancy outcomes. The overall discrimination of the model was presented as the area under the receiver operating characteristic curve (AUROC). Overall performance was assessed using the Brier score.

**Results:**

From 350 pregnant patients (median age 30 [interquartile range (25.2, 35.0)] years-old]), 11.1% had hypertensive disorders, 19.7% required mechanical ventilation support and 6.0% died. The AUROC for in-hospital mortality and for the composite outcome were 0.809 (95% IC: 0.641–0.944) and 0.704 (95% IC: 0.617–0.792), respectively, with good overall performance (Brier = 0.0384 and 0.1610, respectively). Calibration was good for the prediction of in-hospital mortality, but poor for the composite outcome. Women who died had a median age 4 years-old higher, higher frequency of hypertensive disorders (38.1% vs. 9.4%, *p* < 0.001) and obesity (28.6% vs. 10.6%, *p* = 0.025) than those who were discharged alive, and their newborns had lower birth weight (2000 vs. 2813, *p* = 0.001) and five-minute Apgar score (3.0 vs. 8.0, *p* < 0.001).

**Conclusions:**

The ABC_2_-SPH score had good overall performance for in-hospital mortality and the composite outcome mechanical ventilation and in-hospital mortality. Calibration was good for the prediction of in-hospital mortality, but it was poor for the composite outcome. Therefore, the score may be useful to predict in-hospital mortality in pregnant patients with COVID-19, in addition to clinical judgment. Newborns from women who died had lower birth weight and Apgar score than those who were discharged alive.

**Supplementary Information:**

The online version contains supplementary material available at 10.1186/s12884-022-05310-w.

## Background

Coronavirus disease 2019 (COVID-19) has quickly spread worldwide with higher morbidity and lethality than other coronaviruses [1], threatening people’s lives, and more severely the most vulnerable or those under adverse social contexts [2, 3]. Pregnancy imposes physiological adaptations, including modulations of the immune system, which have important implications on the prognosis of viral conditions [4–6]. Most women can experience mild or asymptomatic disease [7], with fatal consequences ranging between 0 to 15.6% among the studies [6, 8–13]. Even in a largely asymptomatic population, COVID-19 has been shown to be associated with maternal inflammatory responses in the maternal-fetal junction and at the circulation [14].

Current studies have shown that pregnant women may be particularly vulnerable to COVID-19 infection [15], as well as developing critical disease and mortality [12, 16–18], which is cause of great concern. Direct and indirect effects of pandemics over pregnancy became a global challenge, changing many aspects of motherhood, mostly in low- and medium-income countries [19]. Large meta-analyses have shown that pregnant women with COVID-19 have higher risk of worse perinatal outcomes, higher requirement of intensive care unit (ICU) admission and invasive mechanical ventilation support, when compared to non-pregnant women with COVID-19 [7].

Furthermore, pregnant women with comorbidities, such as diabetes, hypertensive diseases, heart disease, and lung diseases seem to be more susceptible to severe/critical forms of COVID-19 and maternal mortality [8, 9, 20]. In fact, the literature indicates other risk factors for adverse outcomes, in addition to pre-existing medical conditions, such as older age, being overweight or obese, and being a member of a black or ethnic minority ethnic group [8]. As there are several physiological changes during pregnancy, the development of rapid scoring systems for prognosis applicable for this population is challenging [21].

In Brazil, a country severely hit by the pandemic, COVID-19 became the first cause of maternal death. Therefore, the assessment of clinical characteristics and outcomes in pregnant women who are hospitalized with COVID-19, as well as the factors potentially associated with adverse maternal outcomes in those patients, is of utmost importance for public health [22, 23]. However, there are specificities in clinical parameters in pregnant women, that makes it impossible to use the same scores developed for the non-pregnant without previous assessment.

Therefore, our aim was to assess the performance of a prognosis score, developed and validated for general hospitalized adults (men and women) with COVID-19 in Brazil, to predict in-hospital mortality and mechanical ventilation support in COVID-19 pregnant patients. Additionally, to assess the frequency of adverse pregnancy outcomes, and to compare characteristics of pregnant women who died to those who were discharged.

## Methods

### Study design and participants

The present analysis is a substudy of the Brazilian COVID-19 Registry, an ongoing retrospective multicenter cohort study of consecutive adults both sex patients with laboratory-confirmed COVID-19 patients hospitalized in public and private hospitals in Brazil. The study protocol was published elsewhere [24]. This manuscript adheres to the Strengthening the Reporting of Observational Studies in Epidemiology (STROBE) reporting guideline [25].

**Fig. 1:**
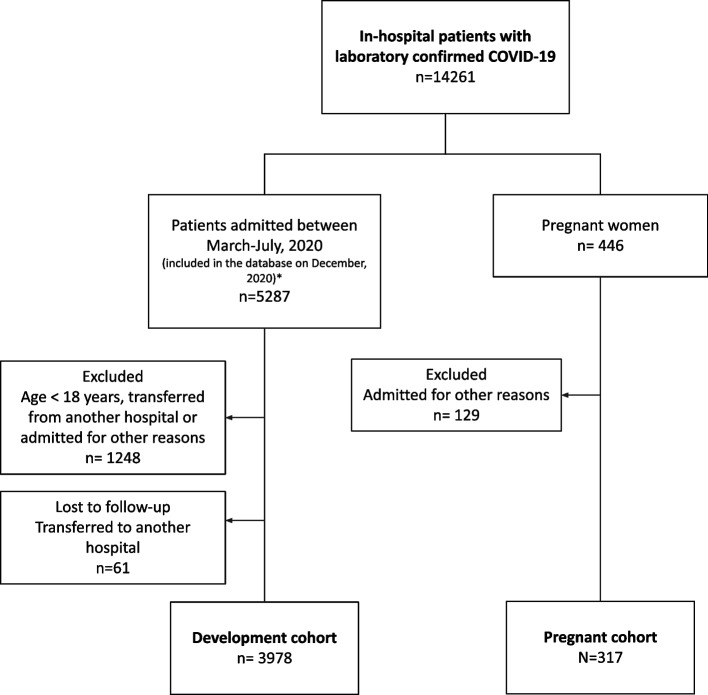
Flowchart of COVID-19 pregnant patients included in the study. *General hospitalized adults (men and women)

For the present analysis, pregnant patients with confirmed COVID-19 admitted to the participating hospitals from April/2020 to March/2022, at any time during the pregnancy, were consecutively enrolled. Patients transferred to hospitals not participating in the Registry without information on final patient outcomes; and those who were admitted to the hospitals due to other conditions, developed symptoms, and had COVID-19 confirmed during hospital admission were not included (Fig. 1).

Patient management was at the discretion of the treating healthcare professionals. Management protocols followed the Brazilian Ministry of Health Guidelines [26, 27].

### Data collection

Study data were collected and managed by trained health professionals using Research Electronic Data Capture (REDCap), hosted at the Telehealth Center of the University Hospital, *Universidade Federal de Minas Gerais* [28, 29]. Clinical characteristics, laboratory data, and obstetric characteristics at admission, as well as events that occurred during the hospital stay and patient outcomes were collected from medical records. Obstetric data were gestational age, pregnancy complications at admission, whether there was delivery and, if so, mode of delivery, birth weight, five-minute Apgar score, and vital state of the newborn. The study protocol and a coding manual guiding data collection with details was agreed with the network of researchers [24]. Furthermore, over the pandemic, the management protocols were updated regularly, following the Brazilian Ministry of Health Guidelines on the management of patients with COVID-19. All patient charts were reviewed thoroughly to confirm the accuracy of the data [24].

### The prognosis score ABC_2_-SPH

Our group previously developed and validated a prognostic scoring model for in-hospital mortality for COVID-19 patients, based on comorbidities, clinical characteristics and laboratory findings at hospital presentation, named the ABC_2_-SPH score [30]. In brief, it has seven variables: age, blood urea nitrogen values, comorbidities, C-reactive protein, peripheral oxygen saturation to fraction of inspired oxygen ratio (SF ratio), platelet count and heart rate, detailed in Table S1 (see Additional file 1). Score development and validation followed strict methodological criteria [31]. It is the only score validated for the Brazilian population, and it has shown high discriminatory ability (AUROC 0.844, 95% CI 0.829 to 0.859), higher than other existing scores [30].

**Fig. 2:**
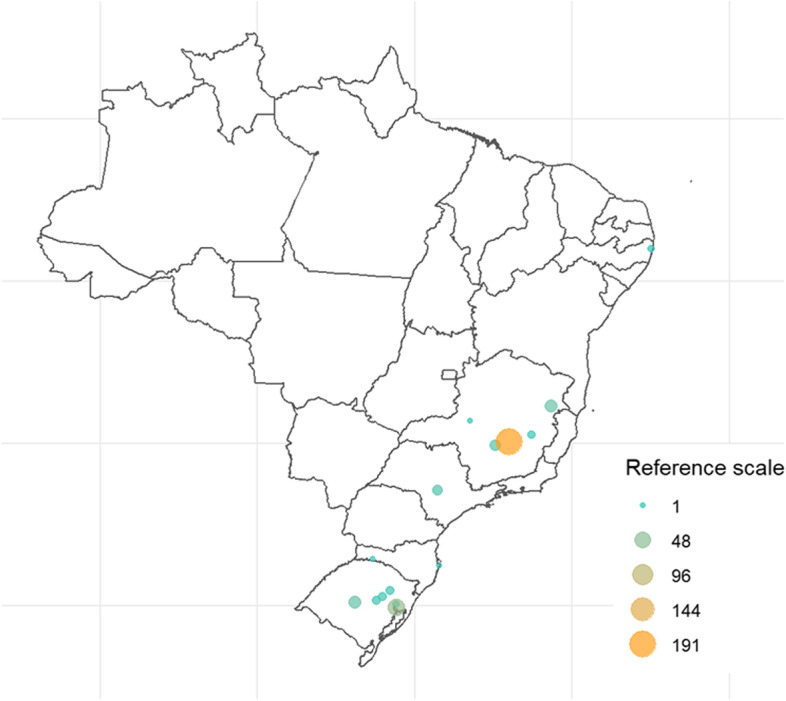
Cities of the hospital of pregnant patients included in this study. *R Core Team (R version 4.0.2). https://www.R-project.org/

After exclusion criteria, 350 pregnant women were identified in 24 centers, in 15 different cities from five Brazilian states (Fig. 2).

### Outcomes

The primary endpoints were in-hospital mortality, and the composite outcome of mechanical ventilation support and in-hospital mortality. Secondary endpoints included pregnancy complications: abortion, ectopic pregnancy, preeclampsia, eclampsia, HELLP Syndrome, abnormal bleeding in childbirth or puerperium, hysterectomy, and puerperal infection.

### Statistical analysis

Descriptive analysis was performed concerning frequency, variability, and central tendency measures. Continuous variables were summarized using medians and interquartile range (IQR), whereas counts and percentages were used for categorical variables. For comparisons between pregnant women who died or were discharged alive, the Chi-squared test or Fisher test was used for the independence hypothesis, and the Mann–Whitney test compared the numerical variables between the groups. A *p*-value lower than 0.05 was considered statistically significant.

Overall performance of ABC_2_-SPH [30] score was evaluated using the Brier score [32]. Calibration was assessed graphically by plotting the predicted outcome of interest (in-hospital mortality or the composite outcome) probabilities against the observed outcome, testing intercept equals zero and slope equals one. The area under the receiver operating characteristic curve (AUROC) described the model’s discrimination. For this, the numeric variable from the score for each pregnant woman was used to predict in-hospital mortality and the composite outcome. Confidence intervals (95% CI) for AUROC were obtained through 2000 bootstrap samples.

We also calculated accuracy, sensitivity and specificity of the ABC2SPH, as well as comparison with other existing scores for the general population [33–37].

All statistical analyses, calibration, and plottings were performed with R software (version 4.0.2) with the tidyverse, pROC, rms packages.

## Results

Clinical characteristics and laboratory findings of the 350 pregnant women are shown in Table 1, and the geographic location of the hospital they were admitted at is shown in Fig. 2. The median age was 30.0 (IQR 25.2, 35.0) years-old, and the majority of them had no previous comorbidities (76.9%). Obesity (11.7%), diabetes (9.1%), and hypertension (11.1%) were the most prevalent underlying medical conditions. Sixty-eight pregnant women needed mechanical ventilation (19.7%), and 21 (6.0%) died. Only three of those who died were not on mechanical ventilation support.

**Table 1:** Demographic, clinical characteristics, and laboratory exams upon hospital presentation of the pregnant patients included in the validation analysis

Characteristics	Overall		Death		Hospital discharge		***p***-value^*^
	Statistic	***n*** = 350^***a***^	Statistic	***n*** = 21^***a***^	Statistic	***n*** = 329^***a***^	
**Age (years)**	30.0 (25.2, 35.0)	350 (100%)	34.0 (28.0, 38.0)	21 (100%)	30.0 (25.0, 35.0)	329 (100%)	0.014
**Comorbidities**							
Hypertension	39 (11.1%)	350 (100%)	8 (38.1%)	21 (100%)	31 (9.4%)	329 (100%)	< 0.001
Diabetes mellitus	32 (9.1%)	350 (100%)	3 (14.3%)	21 (100%)	29 (8.8%)	329 (100%)	0.424
Obesity (BMI ≥ 30 kg/m^2^)	41 (11.7%)	350 (100%)	6 (28.6%)	21 (100%)	35 (10.6%)	329 (100%)	0.025
**Symptoms**^***b***^							
None	14 (4.0%)	350 (100%)	0 (0.0%)	21 (100%)	14 (4.3%)	329 (100%)	> 0.999
Adynamic	40 (11.4%)	350 (100%)	3 (14.3%)	21 (100%)	37 (11.2%)	329 (100%)	0.720
Ageusia	44 (12.6%)	350 (100%)	1 (4.8%)	21 (100%)	43 (13.1%)	329 (100%)	0.493
Anosmia	60 (17.1%)	350 (100%)	4 (19.0%)	21 (100%)	56 (17.0%)	329 (100%)	0.768
Headache	94 (26.9%)	350 (100%)	4 (19.0%)	21 (100%)	90 (27.4%)	329 (100%)	0.563
Rhinorrhea	76 (21.7%)	350 (100%)	3 (14.3%)	21 (100%)	73 (22.2%)	329 (100%)	0.586
Diarrhea	20 (5.7%)	350 (100%)	1 (4.8%)	21 (100%)	19 (5.8%)	329 (100%)	> 0.999
Dyspnea	195 (55.7%)	350 (100%)	13 (61.9%)	21 (100%)	182 (55.3%)	329 (100%)	0.717
Sore throat	35 (10.0%)	350 (100%)	1 (4.8%)	21 (100%)	34 (10.3%)	329 (100%)	0.708
Fever	166 (47.4%)	350 (100%)	8 (38.1%)	21 (100%)	158 (48.0%)	329 (100%)	0.510
Hyporexia	16 (4.6%)	350 (100%)	1 (4.8%)	21 (100%)	15 (4.6%)	329 (100%)	> 0.999
Myalgia	118 (33.7%)	350 (100%)	11 (52.4%)	21 (100%)	107 (32.5%)	329 (100%)	0.103
Nausea/vomiting	43 (12.3%)	350 (100%)	3 (14.3%)	21 (100%)	40 (12.2%)	329 (100%)	0.732
Productive cough	164 (46.9%)	350 (100%)	9 (42.9%)	21 (100%)	155 (47.1%)	329 (100%)	0.878
**Clinical presentation**							
Glasgow coma score = 15	318 (100.0%)		14 (100.0%)		304 (100.0%)		
Respiratory rate (irpm)	22.0 (19.0, 26.2)	252 (72%)	23.0 (19.0, 32.5)	12 (57%)	22.0 (19.0, 26.0)	240 (73%)	0.394
SF ratio	457.1 (405.5, 466.7)	298 (85%)	333.0 (98.2, 456.0)	14 (67%)	457.1 (419.0, 466.7)	284 (86%)	0.002
Heart rate (bpm)	100.0 (88.0, 111.0)	311 (89%)	102.0 (87.5, 111.5)	15 (71%)	100.0 (88.0, 110.5)	296 (90%)	0.945
Systolic blood pressure		314 (90%)		15 (71%)		299 (91%)	> 0.999
≥ 90 (mmHg)	305 (97.1%)		15 (100.0%)		290 (97.0%)		
Inotrope requirement	3 (1.0%)				3 (1.0%)		
Diastolic blood pressure		313 (89%)		15 (71%)		298 (91%)	0.248
≤ 60 (mmHg)	76 (24.3%)		1 (6.7%)		75 (25.2%)		
Inotrope requirement	3 (1.0%)				3 (1.0%)		
**Laboratory exams**							
Hemoglobin (g/L)	11.7 (10.8, 12.6)	297 (85%)	11.9 (11.2, 12.4)	14 (67%)	11.7 (10.8, 12.6)	283 (86%)	0.534
Platelet count (109/L)	199,000.0 (162,000.0, 250,500.0)	295 (84%)	224,500.0 (170,500.0, 265,500.0)	14 (67%)	198,000.0 (162,000.0, 250,000.0)	281 (85%)	0.447
NL ratio	5.3 (3.5, 8.4)	294 (84%)	8.1 (5.5, 12.5)	14 (67%)	5.3 (3.5, 8.3)	280 (85%)	0.034
Lactate value	1.1 (0.8, 1.5)	143 (41%)	1.2 (1.1, 1.4)	10 (48%)	1.1 (0.8, 1.5)	133 (40%)	0.557
C reactive protein (mg/L)	47.0 (18.5, 88.0)	249 (71%)	47.9 (18.9, 90.6)	10 (48%)	47.0 (18.5, 86.1)	239 (73%)	0.750
Urea (mg/dL)	13.3 (10.0, 17.6)	231 (66%)	9.8 (8.0, 18.5)	10 (48%)	13.3 (10.0, 17.3)	221 (67%)	0.324
Creatinine (mg/dL)	0.6 (0.5, 0.7)	255 (73%)	0.5 (0.4, 0.7)	11 (52%)	0.6 (0.5, 0.7)	244 (74%)	0.571
pH	7.4 (7.4, 7.5)	168 (48%)	7.4 (7.4, 7.4)	9 (43%)	7.4 (7.4, 7.5)	159 (48%)	0.310
Arterial pO_2_	85.0 (70.0, 110.2)	168 (48%)	69.0 (52.0, 72.0)	9 (43%)	86.0 (71.3, 111.0)	159 (48%)	0.004
Arterial pCO_2_	29.0 (25.5, 31.0)	169 (48%)	28.9 (28.4, 31.0)	9 (43%)	29.0 (25.3, 31.0)	160 (49%)	0.188
**Outcomes**							
Intensive care unit	113 (32.3%)	350 (100%)	19 (90.5%)	21 (100%)	94 (28.6%)	329 (100%)	< 0.001
Dialysis	14 (4.0%)	350 (100%)	6 (28.6%)	21 (100%)	8 (2.4%)	329 (100%)	< 0.001
Mechanical ventilation	68 (19.7%)	346 (99%)	18 (85.7%)	21 (100%)	50 (15.4%)	325 (99%)	< 0.001
Intra-hospital mortality	21 (6.0%)	350 (100%)	21 (100.0%)	21 (100%)	0 (0.0%)	329 (100%)	< 0.001

*BMI* body mass index, *HCO*_*3*_^*−*^ bicarbonate, *NL ratio* neutrophils-to-lymphocytes ratio, *pH* hydrogen potential, *pCO*_*2*_ carbon dioxide partial pressure, *pO*_*2*_ oxygen partial pressure, *SpO*_*2*_*/FiO*_*2*_ *ratio* peripheral oxygen saturation to fraction of inspired oxygen ratio

^*^Statistical tests performed: Wilcoxon rank-sum test; Fisher’s exact test; chi-square test of independence

^a^Statistics presented: Median (IQR); n (%)

^b^There was no patient with neurological symptoms, arthralgia or skin rash

**Fig. 3:**
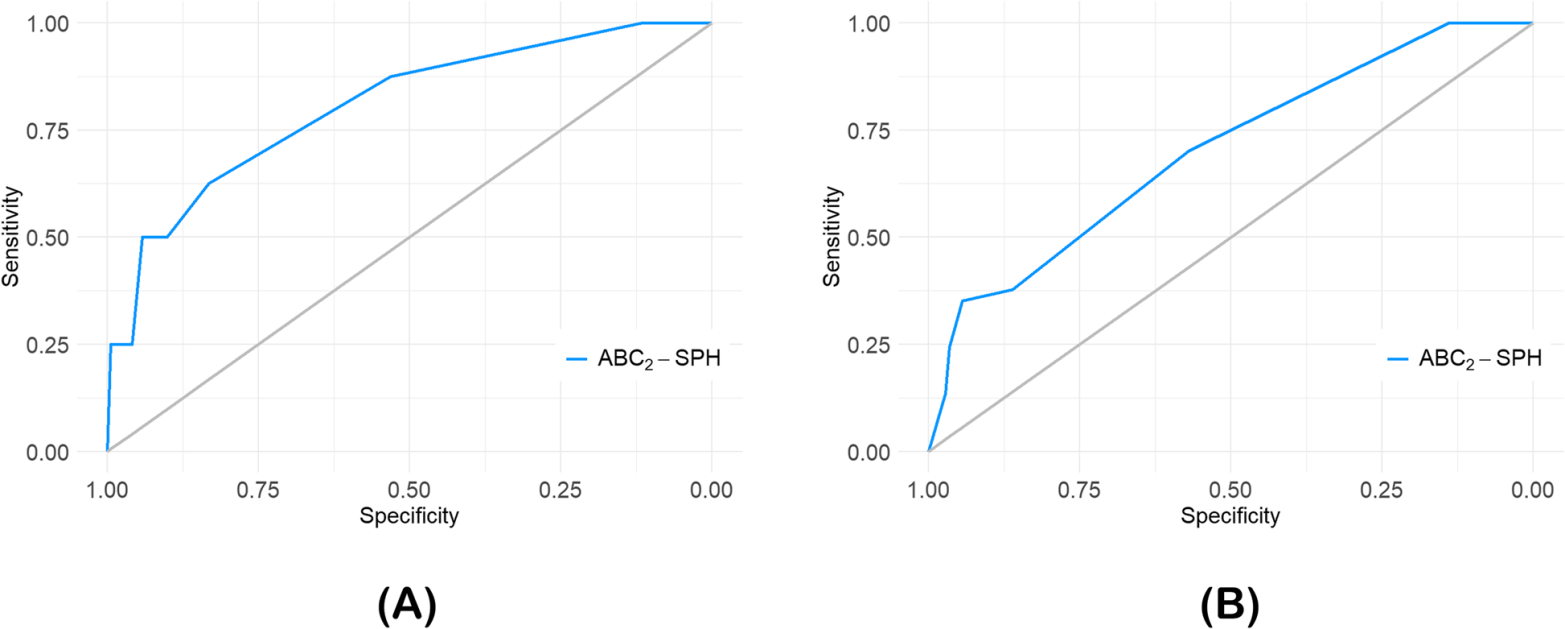
Discrimination of ABC_2_-SPH Score in the sample of pregnant patients to predict in-hospital mortality (**A**), and composite of mechanical ventilation support and in-hospital mortality (**B**)

**Fig. 4:**
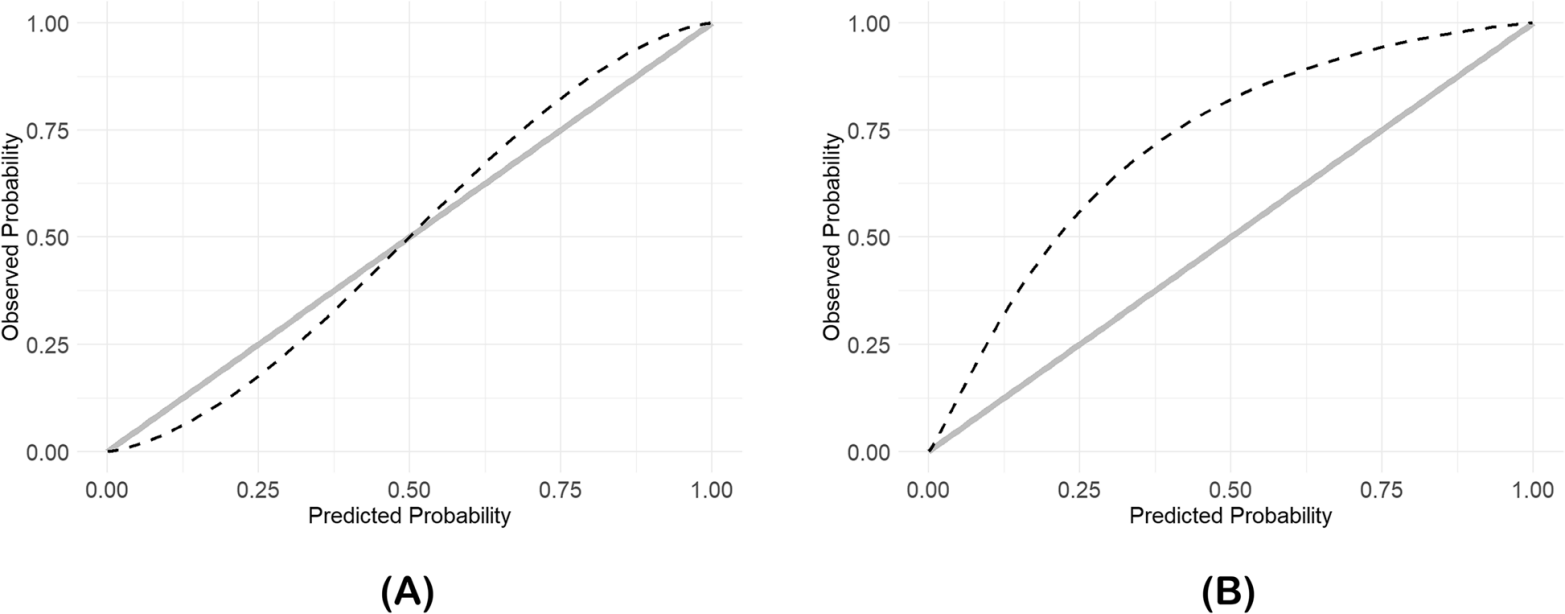
Calibration plot of ABC_2_-SPH Score for in-hospital mortality (**A**), and composite of mechanical ventilation and in-hospital mortality (**B**), for each quartile of pregnant women risk. *It plots the observed and expected death, and the diagonal line represents a perfect agreement between observed and expected probability of the outcome

The ABC_2_-SPH score was able to identify high-risk pregnant women. The area under the ROC curve [AUROC] for in-hospital mortality was 0.809 (95% IC: 0.641–0.944) and for the composite outcome was 0.704 (95% IC: 0.617–0.792) (Fig. 3A and B), with good overall performance (Brier = 0.0384 and 0.1610, respectively). Calibration was also good for the prediction of in-hospital mortality, but it was poor for the composite outcome (Fig. 4A and B). Table S2 shows the comparison between ABC_2_-SPH and other scores (see Additional file 2).

Women who died had a median age 4 years-old higher than those who were discharged alive, as well as a higher frequency of hypertensive disorders (38.1% vs. 9.4%, *p* < 0.001) and obesity (28.6% vs. 10.6%, *p* = 0.025). Dyspnea (55.7%), fever (47.4%), productive cough (46.9%), myalgia (33.7%), and headache (26.9%) were the most frequent symptoms, and the frequency of symptoms was similar between those who died and those who were discharged alive. With regards to clinical presentation and laboratory analysis upon hospital admission, patients who died had a significantly lower median of the SpO_2_/FiO_2_ ratio (333.0 vs 457.1, *p* = 0.002), higher median neutrophils-to-lymphocytes ratio (8.1 vs. 5.3, *p* = 0.034), and lower partial pressure of oxygen (69.0 vs 86.0, *p* = 0.004) than those who were discharged alive (Table 1).

Concerning obstetric characteristics (Table 2), the median gestational age was 31.0 (IQR 24.0, 36.0) weeks overall, and there was no difference between groups. However, those who died had a higher frequency of gestational complications (57.1% vs. 27.3%, *p* = 0.008).

**Table 2:** Characteristics of the pregnant patients who gave birth during COVID-19 hospital stay and their newborns

Characteristics	Overall		Death		Hospital discharge		***P*** value^*^
	Statistic^**a**^	***n*** = 350 pregnant patients / 140 newborns	Statistic^**a**^	***n*** = 21 pregnant patients / 14 newborns	Statistic^**a**^	***n*** = 329 pregnant patients / 126 newborns	
Gestational age (weeks)	31.0 (24.0, 36.0)	348 (99%)	29.0 (25.0, 33.0)	21 (100%)	31.0 (24.0, 36.0)	327 (99%)	0.507
Gestational hypertensive disorder	42 (12.0%)	350 (100%)	4 (19.0%)	21 (100%)	38 (11.6%)	329 (100%)	0.298
Gestational complication^b^	101 (29.1%)	347 (99%)	12 (57.1%)	21 (100%)	89 (27.3%)	326 (99%)	0.008
Vaginal birth or C-Section		139 (40%)		14 (67%)		125 (38%)	0.552
C-section	97 (69.8%)		11 (78.6%)		86 (68.8%)		
Vaginal birth	42 (30.2%)		3 (21.4%)		39 (31.2%)		
Childbirth	139 (40.1%)	347 (99%)	14 (66.7%)	21 (100%)	125 (38.3%)	326 (99%)	0.019
Born alive	125 (91.2%)	137 (98%)	13 (92.9%)	14 (100%)	112 (91.1%)	123 (98%)	> 0.999
Birth weight (grams)	2760.0 (2120.0, 3236.5)	95 (68%)	2000.0 (1250.0, 2150.0)	9 (64%)	2812.5 (2428.5, 3310.5)	86 (68%)	0.001
Apgar Score	8.0 (6.0, 9.0)	105 (75%)	3.0 (2.0, 5.0)	9 (64%)	8.0 (7.0, 9.0)	96 (76%)	< 0.001

*Statistical tests performed: Fisher’s exact test; Wilcoxon rank-sum test

^**a**^Statistics presented: n (%); Median (IQR)

^b^Abortion, hypertensive disturbances, diabetes, preterm birth

Among 350 pregnant women, 139 (40.1%) gave birth during the in-hospital stay. One woman delivered twins, totaling 140 newborns. Of those, 125 (91.2%) were alive at hospital discharge. Birth weight in grams (2000 vs 2813, *p* = 0.001) and five-minute Apgar score (3.0 vs 8.0, *p* < 0.001) were lower in newborns from pregnant women who died from COVID- 19 when compared to those who were discharged alive (Table 2).

## Discussion

The main contribution of the present analysis was to validate the ABC_2_-SPH score in 350 pregnant women from 24 Brazilian hospitals. The ABC_2_-SPH score has been shown to be a reliable tool in estimating in-hospital mortality risk in pregnant COVID-19 patients. In this population, the score had good overall performance for the primary outcomes and good discriminatory ability. Calibration was good for the prediction of in-hospital mortality, but it was poor for the composite outcome of in-hospital mortality and mechanical ventilation support. The score is simple, objective, uses variables easily available at hospital presentation and it may be easily calculated. Model performance comparison surpassed other existing scores, commonly used in the general population.

In fact, assessing predictors of critical outcomes in COVID-19 may advise timely treatments and better prepare facilities to overcome extra adversities during pregnancy. Our findings support the employment of the score as a tool in estimating in-hospital mortality at admission in pregnant patients. Therefore, it is of utmost importance to take into account that the score should be used in addition to the clinical judgment, to support clinical decision, for example, to help screening pregnant women who need more frequent reassessments, as well as to help to assess which one to refer to intensive care, in cases of limited resources. As a screening tool, it is of utmost importance to have a high sensitivity, to avoid missing as few cases as possible. In the present analysis, ABC_2_-SPH achieved 96.0% sensitivity, with a very precise confidence interval (91.8–98.4%), higher than any other score tested.

On the contrary, our findings evidence against the use of the score to predict the composite outcome of mechanical ventilation support and in-hospital mortality. In a recent analysis from our group (data not published yet), ABC_2_-SPH score did not have good overall performance to predict mechanical ventilation support in general (non-pregnant) patients. Therefore, the present results may reflect the fact that the score is not a good predictor for mechanical ventilation overall.

Several prediction scores have been proposed for use in the nonpregnant population with COVID-19 with varied success. The study conducted by Jones et al. (**2021**) [38] validated the 4C score for Canadian patients obtaining an AUC of 0.770 (95% CI 0.790–0.870). In addition, the accuracy of this prediction model (4C Mortality), beyond NEWS and CURB-65 was compared among the Romanian population with AUC of 0.818 (95% CI 0.718–0.919), 0.861 (95% CI 0784–0.939), and 0.801 (95% CI 0.681–0.922), respectively [39, 40]. In the present study, we tested these aforementioned scores, together with other scores commonly used for general COVID-19 patients, and ABC_2_-SPH outperformed all of them.

As aforementioned, many clinical parameters of existent scores developed for the general (non-pregnant) population are deeply modified by physiological adaptations of pregnancy. Notably, these adaptations are challenging for using the scores developed for the general population without further validation and can contribute to an understanding of the lack of prediction models for the prognosis of COVID-19 in this population, despite the fact that several prognostic scores have been developed for COVID-19 [41–44]. One multicenter retrospective cohort study including eight hospitals from four countries (*n* = 973) proposed two models to predict ICU admission and maternal death in pregnant women with symptomatic COVID-19 [45], however, both models are limited by methodological bias, with the absence of external (even geographic) validation.

Our study observed high in-hospital mortality in pregnant women (6%). A study based on secondary data from Brazil (975,109 cases) suggested that pregnant women with COVID-19 have approximately twice the mortality rates of men and non-pregnant women [46]. Takemoto et al. (**2020**) [16] reported high mortality among Brazilian pregnant women with COVID-19, approximately 20 maternal deaths out of 125,218 overall cases and 8536 deaths (as of May 7, 2020), with lethality of 15.6% in 2021 [12]. Similarly, another study found an association between COVID-19 and worse clinical outcomes for pregnant women in Brazil, with a 3.4 times higher death rate than any other acute respiratory distress syndromes (ARDS) etiologies [15]. According to a systematic review with 2670 patients from seven countries, the differences in results for maternal characteristics reflect the profile of the patient of each country of origin [9]. This study (*n* = 38 studies, 2670 patients, 52.6% from China), have shown a significant variation between maternal age among pregnant women with COVID-19, percentages of C-sections, maternal mortality rate and newborn outcomes [9]. In fact, is it possible that pregnant Brazilian patients have different characteristics from those from other countries, placing them in the leadership of maternal deaths due to COVID-19 worldwide [47]. One of them is the prevalence of underlying diseases, especially preeclampsia and obesity, conditions that are known inflammatory, risk factors for COVID-19 complications. Besides, an important contributor to greater mortality in the country were the barriers to access to prenatal care during the pandemic, inadequate monitoring of obstetric complications and barriers to access intensive care [4, 17, 47–49].

Despite having similar symptoms, our analysis showed differences between pregnant women who died and those who were discharged. Those who died had higher age, prevalence of hypertension, obesity and, as expected, in-hospital complications than the ones who were discharged alive. These findings are consistent with a large study from the Centers for Disease Control and Prevention (CDC), comparing 386,028 positive nonpregnant women in their reproductive age (15–44 years), with 23,434 SARS-CoV-2 positive pregnant women, demonstrating that death occurred more frequently among women aged 35–44 years than among those aged 15–24 years. When stratified by age, all outcomes, such as hospitalization, ICU admission, receipt of mechanical ventilation, and death were more frequently in pregnant women aged 35–44 years than among those aged 15–24 years [50]. Additionally, a brief communication conducted by Takemoto et al. (**2020**) [17] collected the effect of 978 pregnant women with COVID-19 in Brazil, indicating that women who died had higher maternal age (31.5 years). A living systematic review has shown that increasing age (odds ratio 1.83, 95% confidence interval 1.27 to 2.63; seven studies, 3561 women), high body mass index (2.37, 1.83 to 3.07; five studies, 3367 women), any pre-existing maternal comorbidity (1.81, 1.49 to 2.20; 3 studies; 2634 women), chronic hypertension (2.0, 1.14 to 3.48; two studies, 858 women), pre-eclampsia (4.21, 1.27 to 14.0; 4 studies; 274 women), and pre-existing diabetes (2.12, 1.62 to 2.78; 3 studies, 3333 women) are maternal risk factors associated with severe COVID-19 [8]. Non-white ethnicity (1.61, 1.05 to 2.47; 3 studies, 31,469 women; 2.23, 1.25 to 3.97; 1 study, 669 women; respectively) and high body mass index (2.27, 1.20 to 4.31; 3 studies, 31,085 women; 6.61, 1.98 to 22.02; 2 studies, 485 women; respectively; Table 2) were associated with maternal death and the need for invasive ventilation [8].

In the present analysis, the most common laboratory findings among patients who died from COVID-19 were lower median SpO_2_/FiO_2_ ratio (333.0 vs 457.1, *p* = 0.002), higher median neutrophils-to-lymphocytes ratio (8.1 vs 5.3, *p* = 0.034), and lower partial pressure of oxygen (69.0 vs 86.0, *p* = 0.004). During pregnancy, vital signals had proper values, including a slight drop in SpO_2_ [51]. It is important to mention that in this period the circulatory system undergoes physiological changes, starting early in its course, driven by peripheral vasodilatation, increased heart rate and stroke volume, reduced pulmonary vascular resistance, and reduced pulmonary residual capacity. These changes may affect the course of viral infections [52, 53]. Regarding inflammatory markers, the existing evidence is conflicting on whether the pregnancy is an immunological contributor to the severe progression of COVID-19 [54]. Successful pregnancy depends on a responsive immune system, which explains reports of universal COVID-19 testing during pregnancy, that the vast majority is asymptomatic or has mild COVID-19 [54, 55]. The unit maternal and the fetoplacental immune system is responsive, protecting both the mother and the fetus against threats from the environment [56].

Nevertheless, we observed that childbirth had an impact of COVID-19. C-section was performed in 71.0% of childbirths, with lower birth weight in babies of pregnant women who died (low birth weight, 2000 vs 2813, *p* = 0.001), and 13 babies died. An aforementioned systematic review [9] analyzed cesarean delivery rates geographically and found rates to be considerably higher in China (83.9%), followed by the United Kingdom (71.9%), with Spain with the lowest rate of C-sections (35.9%). The reasons for these practices are unclear, but it may be attributable to the habitual medical practice of each country, in addition to the lack of guidelines and recommendations at the beginning of the pandemic. Regarding low birth weight, the placenta is a selective barrier able to protect the developing fetus against infections, including SARS-CoV-2 virus infection [57], and it acts as an immunity-modulating organ, regulating immune responses of cells present both at the implantation site and systemically [58]. However, evidence of fetal vascular malperfusion or thrombosis has been observed in COVID-19, which may be related to an exacerbated maternal systemic inflammatory response and hypercoagulable state [59, 60]. In the meantime, our study observed lower median Apgar score in newborns of pregnant women who died from COVID-19, compared to those who were discharged alive (3.0 vs 8.0, *p* < 0.001). In cases of fetal distress, prematurity, and severe/critical maternal disease the Apgar scores are lower [61, 62].

Based on our results, we warn against the use of non-pregnant COVID-19 prognosis scores in pregnant patients to predict adverse outcomes without proper validation. While the control of COVID-19 pandemic is still challenging in many places, fast and efficient assessment of the prognosis of the COVID-19 is of utmost importance. We can expect the downstream effects of COVID-19 to be apparent for a number of years. Further studies with large sample sizes are required for the development and validation of a more accurate model to predict other adverse outcomes, such as mechanical ventilation and pregnancy outcomes, concerning the specificities of pregnant patients affected by COVID-19. Scores for pregnant women would be useful for early identification of cases at higher risk of worse outcomes in this highly vulnerable group of women. Further studies are also necessary to identify risks in pregnancy-related critical illness [21] due to COVID-19 or other causes. Evidence-based modeling could provide a proper prognosis score assessment tool that will help guide decision-making, develop patient care plans, and better allocate resources.

Even with its multiple strengths, the present study has some limitations. Recalibration of the ABC_2_-SPH score may improve its prediction of the effects of COVID-19 on pregnant women. However, our sample size is not large enough for this analysis (at least 100 events for recalibration) [31]. This is a topic for future studies with larger sample sizes. Additionally, details about diagnosis of hypertensive syndromes of pregnancy, subtypes of diabetes during pregnancy may differ among the perinatal centers involved in this cohort. Besides, the study missed details to specify gestational complications. The newborn data was used to infer possible complications related to pregnancy and delivery, the analysis of newborn outcomes secondary to COVID-19 requires a different study design and was not the purpose of the present analysis. In fact, this is a topic for an ongoing study from our group. Additionally, we have not investigated the impact of each individual SARS-CoV-2 variant on pregnant women. Different variants had different rates of adverse obstetric outcomes and different prognosis [63]. Lastly, due to the exclusion of pregnant/lactating women from the preliminary vaccine trials [64], the Brazilian vaccination campaign for pregnant women started in July 2021, and our sample size did not allow a stratified analysis. Further studies are required on both topics.

## Conclusions

This study has shown that the ABC_2_-SPH score, developed in Brazilian general patients, was not able to sufficiently identify adverse clinical outcomes in pregnant patients with COVID-19.

We warn against the use of prediction models for general inpatients COVID-19 prognosis in pregnant women. Further studies with large sample sizes are required for the development and validation of a more accurate model to predict poor outcomes, concerning the specificities of pregnant patients affected by COVID-19.

## Supplementary Information

**Additional file 1: Table S1.** ABC2-SPH score for in-hospital mortality in patients with COVID-19*.

**Additional file 2: Table S2.** Discrimination of risk scores within validation cohort (complete cases).

## Data Availability

All data generated or analyzed during this study are included in this published article and its supplementary information files.

## Abbreviations

ARDS: Acute respiratory distress syndrome
AUROC: Receiver Operating Characteristic Curve
BMI: Body mass index
CDC: Centers for Disease Control and Prevention
COVID-19: Coronavirus Disease 2019
HCO_3_^−^: Bicarbonate
ICU: Intensive care unit
IQR: Interquartile Ranges
NL ratio: Neutrophils-to-lymphocytes ratio
pCO_2_: Carbon dioxide partial pressure
pH: Hydrogen potential
pO_2_: Oxygen partial pressure
REDCap: Research Electronic Data Capture
SARS-CoV-2: Severe Acute Respiratory Syndrome Coronavirus 2
SF ratio: Peripheral oxygen saturation to fraction of inspired oxygen ratio
SpO_2_: Peripheral Oxygen Saturation
STROBE: Strengthening the Reporting of Observational Studies in Epidemiology
