# Supplementary material for: Mechanical ventilation and death in pregnant patients admitted for COVID-19: a prognostic analysis from the Brazilian COVID-19 registry score
Source: BMC Pregnancy Childbirth. 2023 Jan 10;23:18. doi: 10.1186/s12884-022-05310-w (PMC9830611; doi:10.1186/s12884-022-05310-w)
Supplement: Supplementary file 1 — Additional file 1: Table S1. ABC2-SPH score for in-hospital mortality in patients with COVID-19*. [file 12884_2022_5310_MOESM1_ESM.docx]

**SUPPLEMENTARY MATERIAL**

| **Table S1.** ABC2-SPH score for in-hospital mortality in patients with COVID-19*. | | |
| --- | --- | --- |
|  | **Variables** | **ABC_2_-SPH score** |
| **A** | Age (years)  < 60  60 - 69  70 - 79  ≥ 80 | 0  1  3  5 |
| **B** | Blood urea nitrogen (mg/dL)**  < 42  ≥ 42 | 0  3 |
| **C_2_** | Comorbidities  0 - 1  ≥ 2 | 0  1 |
|  | C-reactive protein (mg/dL)  < 100  ≥ 100 | 0  1 |
| **S** | SpO_2_/FiO_2_ ratio | 0  1 |
| **P** | Platelets count (x10^9^/L)  > 150  100 - 150  ≥ 100 | 0  1  2 |
| **H** | Heart rate (bpm)  ≤ 90  91 - 130  ≥ 131 | 0  1  2 |
| **Marcolino MS, Ziegelmann PK, Souza-Silva MVR, do Nascimento IJB, Oliveira LM, Monteiro LS, et al.* Clinical characteristics and outcomes of patients hospitalized with COVID-19 in Brazil: results from the Brazilian COVID-19 Registry. Int J Infect Dis. 2021;11(21):00030-8.  **When converted to urea, the cut-off is 90 mg/dL | | |
