# Supplementary material for: Mechanical ventilation and death in pregnant patients admitted for COVID-19: a prognostic analysis from the Brazilian COVID-19 registry score
Source: BMC Pregnancy Childbirth. 2023 Jan 10;23:18. doi: 10.1186/s12884-022-05310-w (PMC9830611; doi:10.1186/s12884-022-05310-w)
Supplement: Supplementary file 2 — Additional file 2: Table S2. Discrimination of risk scores within validation cohort (complete cases). [file 12884_2022_5310_MOESM2_ESM.docx]

**SUPPLEMENTARY MATERIAL**

**Table S2.** Discrimination of risk scores within validation cohort (complete cases)

| **Score** | **Number of patients** | **Number of death (%)** | **AUROC (95% CI)** | **Accuracy (95% CI)** | **Sensitivity (95% CI)** | **Especificity (95% CI)** |
| --- | --- | --- | --- | --- | --- | --- |
|  |  |  |  |  |  |  |
| **ABC_2_-SPH** | 181 | 8 (4.4%) | 0.817 (0.641-0.944) | 0.939 (0.894-0.969) | 0.960 (0.918-0.984) | 0.500 (0.157-0.843) |
| **4C Mortality Score** | 166 | 6 (3.6%) | 0.658 (0.391-0.879) | 0.639 (0.56-0.712) | 0.637 (0.558-0.712) | 0.667 (0.223-0.957) |
| **NEWS2** | 99 | 4 (4.0%) | 0.639 (0.362-0.921) | 0.838 (0.751-0.905) | 0.853 (0.765-0.917) | 0.500 (0.068-0.932) |
| **A-DROP** | 146 | 8 (5.5%) | 0.638 (0.466-0.818) | 0.76 (0.683-0.827) | 0.775 (0.697-0.842) | 0.500 (0.157-0.843) |
| **NEWS-FAST** | 173 | 9 (5.2%) | 0.507 (0.319-0.679) | 0.699 (0.625-0.767) | 0.72 (0.644-0.787) | 0.333 (0.075-0.701) |
| **CURB 65** | 194 | 8 (4.1%) | 0.502 (0.331-0.701) | 0.907 (0.857-0.944) | 0.941 (0.897-0.97) | 0.125 (0.003-0.527) |
